# Review of Thermoresponsive Electroactive and Magnetoactive Shape Memory Polymer Nanocomposites

**DOI:** 10.1021/acsomega.2c05930

**Published:** 2022-11-02

**Authors:** Clara Pereira Sánchez, Christine Jérôme, Ludovic Noels, Philippe Vanderbemden

**Affiliations:** † Department of Electrical Engineering and Computer Science, University of Liège, Liège 4000, Belgium; ‡ CERM, CESAM-RU, University of Liège, Liège 4000, Belgium; § Department of Aerospace and Mechanical Engineering, University of Liège, Liège 4000, Belgium

## Abstract

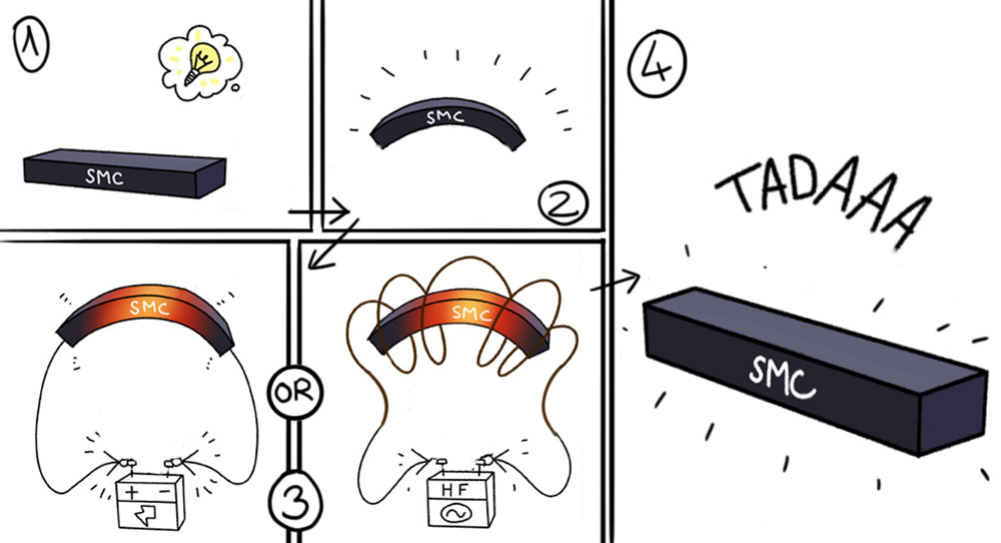

Electroactive and magnetoactive shape memory polymer nanocomposites (SMCs) are multistimuli-responsive smart materials that are of great interest in many research and industrial fields. In addition to thermoresponsive shape memory polymers, SMCs include nanofillers with suitable electric and/or magnetic properties that allow for alternative and remote methods of shape memory activation. This review discusses the state of the art on these electro- and magnetoactive SMCs and summarizes recently published investigations, together with relevant applications in several fields. Special attention is paid to the shape memory characteristics (shape fixity and shape recovery or recovery force) of these materials, as well as to the magnitude of the electric and magnetic fields required to trigger the shape memory characteristics.

## 1 Introduction

Shape memory polymers (SMPs) and their composites (SMCs) are smart materials that are able to respond to certain external stimuli by changing their shape. In most cases, these shape memory materials (SMMs) are thermoresponsive, meaning that their shape memory properties are triggered depending on their temperature. Other response methods exist such as light, pH, and solvents,^1^ but these will not be covered in this review. The simplest shape memory cycle of a thermoresponsive SMM is composed of a shape programming step and a shape recovery step. The shape programming step starts with the SMM in its original shape, commonly termed the permanent shape, that is heated above its transition temperature (*T*_trans_). The *T*_trans_ values of a SMM are those related to the phase transitions that control the shape memory process: i.e., the glass transition or the melting–crystallization transition. Once at a temperature passes *T*_trans_, a stress is applied, leading to a deformation from the permanent shape to the desired temporary shape. The shape programming step finishes by cooling the material under stress below *T*_trans_ while maintaining said deformation in order to fix the temporary shape, after which the applied stress can be released. Reheating the material above *T*_trans_ leads to the shape recovery from the temporary shape to the permanent shape due to stress relaxation.^2^

Conventionally, the shape recovery is triggered with direct heating by placing the SMM close to a heat source (e.g., inside an oven). This technique, which relies on an external heater, is occasionally impossible, problematic, or even dangerous. In recent years, much effort has been put into remotely inducing the shape recovery by indirectly heating the SMM using an electric or a magnetic field.^3^ However, pristine SMPs are not suitable for these remote actuation triggers because of their high electric resistivity and insensitivity to magnetic fields, akin to the properties of conventional polymers. In order to benefit from these alternative heating mechanisms, either high electrical conductivity or magnetic fillers are embedded within the SMPs, hence conforming a SMC. For conciseness, SMM is used in the remainder of the article to refer to SMPs and SMCs.

In this review, we address the topic of shape memory polymer nanocomposites activated by an electric or magnetic field. We start with a brief introduction and overview on the topic of shape memory and the different types of shape memory behavior: one-way, two-way, and multiple shape memory. We continue by addressing the phenomena responsible for electro- and magnetoactivation of SMMs. After this theoretical introduction, we discuss the typical nanofillers used to disperse within the polymer matrix in order to confer electroactive and magnetoactive properties to SMCs. Some of the numerous works published in the past few years are summarized, paying special attention to the shape memory characteristics and the magnitude of the electric or magnetic fields required for activation. As an additional tool for the reader, an extensive table of additional references on these SMMs is available in the Appendix. Some recent progress and applications of these SMCs are discussed next. Finally, future horizons and challenges in the investigation of SMMs are pointed out.

## 2 Basic Concepts on SMPs and Their Composites

The shape memory effect is a result of an adequate polymer molecular architecture including two parts: (i) the netpoints that build up the permanent shape of the SMP and (ii) the molecular-switchable segments that are responsible for fixing the temporary shape of the SMP.^4^ The netpoints are cross-links that can be either chemical (covalent bonds) or physical. The latter is related to interactions among the molecules of the polymer network that consists of crystallites, hydrogen bonds, or ionic interactions. Physically cross-linked netpoints are weaker than their covalent chemical counterpart; thus, thermoplastics are more prone to incomplete shape recovery.^5^ Regarding the switchable segments, they are reversible cross-links that can also be of physical or chemical nature.

Depending on the nature of both the netpoints and the switchable segments, a comprehensive SMP classification consisting of four classes was introduced by the research group of Mather^6^ and since reported by many others.^1,4,7−10^ The classes are (I) glassy covalently cross-linked thermosets, (II) semicrystalline covalently cross-linked polymer networks, (III) glassy physically cross-linked copolymers and blends, and (IV) semicrystalline physically cross-linked block copolymers.

The *T*_trans_ of a SMM is the characteristic temperature related to the formation and destruction of the switchable segments. In general, the *T*_trans_ of semicrystalline SMPs is related to the melting–crystallization phase transition, i.e. the melting temperature (*T*_m_) during heating and the crystallization temperature (*T*_c_) during cooling. On the other hand, the *T*_trans_ of glassy SMPs is related to the glass transition temperature (*T*_g_). These glassy SMPs usually have a broader *T*_trans_ range than semicrystalline-network SMPs and therefore a slower recovery. Although this may be regarded as an undesirable feature, in combination with the biocompatibility of some of these polymer networks, it makes them excellent candidates for biomedical applications.^11−13^ Nevertheless, they are not ideal for applications where a fast shape recovery is needed. On the other hand, traditionally, the majority of covalent bonds are strong chemical cross-links that are stable and hard to break. Thus, most polymers that are covalently cross-linked cannot be recycled or reprocessed. In order to overcome the disadvantages associated with covalent cross-links while still having a strong bond among molecules, dynamic covalent bonds can be incorporated in SMPs. Dynamic covalent bonds are reversible and thus are able to be broken and formed when they are subjected to certain stimuli such as heat, light, or pH. In recent years more and more polymers have been produced incorporating dynamic covalent bonds in order to achieve materials that can self-heal, reshape for 3D printing purposes, and have shape memory.^14^ There is a wide choice of methods to obtain dynamic covalent bonds, and the corresponding literature^14−18^ is available.

### 2.1 The Basic Shape Memory Effect

**Figure 1:**
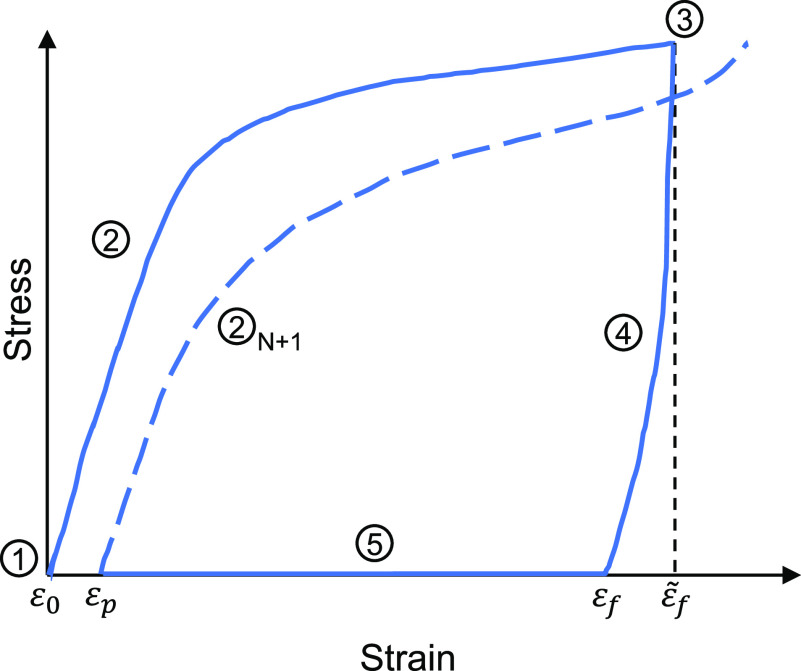
Illustration of the stress vs strain curve of a SMM during two conventional consecutive shape memory cycles performed under uniaxial tension. The marked strain levels *ε*_0_, *ε*_p_, *ε*_f_, and  are used to calculate the shape fixity and shape recovery ratios.

The shape memory effect of a SMM arises after the shape programming process that is achieved in three steps. To aid in the explanation, the stress evolution with applied strain of a SMP is illustrated in Figure 1. The shape programming process starts by ① heating the material, initially in its permanent shape, above a characteristic *T*_trans_ (either *T*_g_ or *T*_m_ depending on the polymer). Second, ② a mechanical deformation is applied on the material while it is above *T*_trans_. This mechanical deformation leads to a strain denoted by  in Figure 1. Finally, the programming process finishes by ③ cooling the material below *T*_trans_ while maintaining the desired load or the desired deformation. ④ Unloading the sample toward zero stress may result in a fixed strain *ε*_f_ slightly lower than . After this programming process is finished, the shape memory material is fixed in its new deformed temporary shape. During the applied deformation, the SMP, with either an amorphous or semicrystalline structure, is in the rubbery state and has a rubber-like behavior. In other words, the SMP above its *T*_trans_ exhibits entropic elasticity. Entropic elasticity exists while deforming an elastomer (or a SMP in the amorphous state) because the high mobility of the polymer chains permits the originally disordered structure to align in the direction of deformation.^19^ Ordering the polymeric chains decreases the entropy of the material, and this energetic state gets fixed by vitrification or crystallization upon cooling the material below *T*_trans_.

During the shape memory programming step, the shape memory material has stored energy due to the freezing of the lower entropic polymer network. The new temporary deformed shape can be viewed as a metastable shape: when the material is exposed to a certain external stimulus, the stored energy will be released. ⑤ Reheating the SMM above *T*_trans_ will cause a shape change back toward the permanent shape. This process, in which the material recovers the preferred high entropic state, is called shape recovery. Different temporary shapes can be attained by repeating the programming process with a different deformation step. Nevertheless, the primary permanent shape is always approximately recovered during the shape memory cycle. This is because *T*_trans_ only affects the switchable links that are related to the temporary shape, whereas the permanent shape is dictated by the netpoints of the SMM. Any damage on the netpoints during the shape memory cycle, for instance breaking of the netpoints due to excessive deformation in the shape programming process, may cause the recovered permanent shape to slightly differ from the original permanent shape.

Two quantities are commonly used to evaluate the quality of a SMM: the shape fixity (*R*_F_) and shape recovery ratios (*R*_R_). They are calculated using eqs 1 and 2, respectively.
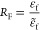
1
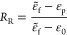
2

The shape fixity ratio quantifies the ability of the SMM to fix the temporary shape and it is calculated as the ratio of strain at the end of ③ cooling (ε_f_ indicated in Figure 1) over the strain at the end of ② elongation ( in Figure 1). On the other hand, the shape recovery ratio gives an idea of how well the SMM “remembers” its permanent shape by relating the strain during shape recovery to the strain due to elongation.

### 2.2 Types of Shape Memory Behavior

**Figure 2:**
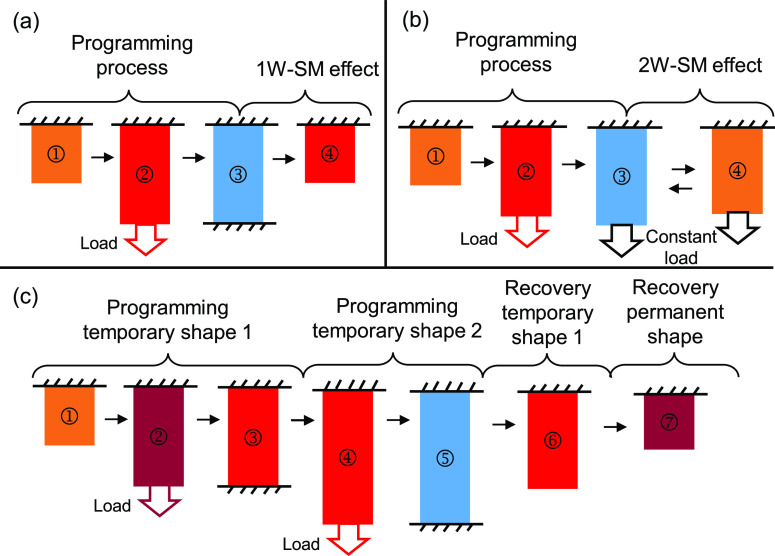
Illustration of the most common shape memory behaviors of SMMs: (a) one-way shape memory effect; (b) two-way shape memory effect; (c) multiple shape memory effect.

The shape memories presented in this section include the simplest one-way shape memory (1W-SM) effect. This section will also define the more complex shape memory behaviors commonly found in the literature, namely the two-way shape memory (2W-SM) effect and the multiple shape memory effect. An illustration of these behaviors is shown in Figure 2, and an explanation is provided next.

#### 2.2.1 One-Way Shape Memory (1W-SM) Effect

The simple 1W-SM effect has already been explained in the previous sections. As illustrated in Figure 2a, it consists of a shape programming process composed by ① heating, ② deformation above *T*_trans_, and ③ fixing of the temporary shape by cooling below *T*_trans_. The programming process is followed by ④ reheating above *T*_trans_ with no load to trigger shape recovery toward the permanent shape. Once the shape recovery has been completed, the SMP is no longer thermoresponsive. To benefit from the shape memory effect again, another shape programming process needs to be performed on the material. Most shape memory polymers and their composites show this type of memory effect. In fact, it was believed until the late 2000s that only shape memory alloys but neither polymers nor their composites could show a two-way shape memory effect. Many examples of SMPs exhibiting the 1W-SM effect can be found in the literature.^20−25^ Furthermore, a more exhaustive review on 1W-SM polymers and their composites has been given by Leng et al.^26^

#### 2.2.2 Two-Way Shape Memory (2W-SM) Effect

The two-way shape memory (2W-SM) effect is present in some semicrystalline SMPs and their composites.^27,28^ A typical 2W-SM effect under tension is illustrated in Figure 2b. The programming process is the same as for the 1W-SM effect: ① heating above *T*_trans_, ② deformation above *T*_trans_, and ③ cooling below *T*_trans_. The 2W-SM effect in semicrystalline polymers arises, in general, by the cyclic ④ heating and ③ cooling above and below the respective *T*_trans_ at a constant load. In this case, the SMP is not left free to deform to the permanent shape but, instead, the shape keeps varying between a primary and a secondary temporary shape. The primary temporary shape is that obtained at the end of ② loading and recovered every time that the SMP is ④ reheated above its *T*_trans_ at constant load. The secondary temporary shape is that obtained by ③ cooling below *T*_trans_ at a constant load. The change in shape between the primary and the secondary temporary shapes can be repeated several times without the need of any other additional programming process.^13^ The 2W-SM effect in semicrystalline polymers under constant tensile stress is characterized by a contraction of the material in the loading direction during heating above *T*_trans_. Conversely, elongation along the loading direction is observed upon cooling below *T*_trans_. Thus, the second temporary shape is longer than the first. Generally, if the reheating above *T*_trans_ happens in a stress-free configuration, the permanent shape is recovered, following the conventional 1W-SM effect.

In 2008, Chung, Romo-Uribe, and Mather^29^ first reported the 2W-SM effect of a semicrystalline network of poly(cyclooctene) under constant stress. They showed that a lower cross-linking density would lead to a higher cooling-induced crystallization and, hence, a wider 2W-SM effect. Furthermore, *T*_m_ and *T*_c_ are found to be shifted to higher values on lowering the crystallization degree.

There is still disagreement on the mechanism behind the 2W-SM effect in semicrystalline polymer networks.^30^ Most authors seem to agree on a melting-induced contraction and a crystallization-induced elongation as the (main) mechanism: oriented crystallites are formed in the direction of loading during cooling.^30,31^ Chung, Romo-Uribe, and Mather postulated that the mechanism was a combination between crystallization-induced elongation and rubber elasticity.^29^

Generally, the 2W-SM effect is shown when the polymer is placed under nonzero stress.^28,30,32−42^ Nevertheless, a few freestanding 2W-SM polymers have also been reported. These reports ascribe the freestanding 2W-SM effect to different sources, including two melting transitions, one broad melting transition, and chemically heterogeneous structures by creating thermostable crystallites after deformation-induced crystallization.^43−48^ Furthermore, other types of materials such as liquid-crystalline elastomers^49,50^ and shape memory composite laminates^51,52^ have been described to exhibit 2W-SM effect.

#### 2.2.3 Multiple Shape Effect

Triple shape memory materials are those that can utilize twice the 1W-SM effect in order to switch from a one temporary shape to another and from the latter to the permanent shape. A conventional 1W-SM effect is based on one *T*_trans_ for shape recovery. In the case of triple SMMs, the two shape changes are related to either one broad temperature interval for the transition or to two separated *T*_trans_ values. This is because a broad thermal transition can be regarded as an infinite amount of abrupt thermal transitions.^53,54^

For a triple SMM, two independent shape programming processes need to be followed. Figure 2c illustrates the typical procedure followed for a triple SMM based on the two transition temperatures *T*_trans,1_ and *T*_trans,2_ such that *T*_0_ > *T*_trans,1_ > *T*_trans,2_, where *T*_0_ is the starting temperature. The deformation to obtain the two different temporary shapes in the illustration is applied by uniaxial tension. The first programming process consists of ① heating the SMM in its permanent shape at *T*_0_ to a temperature above *T*_trans,1_, where the polymer chains gain mobility, and ② the deformation to the first temporary shape can be applied. The deformation produces an orientation of the polymer chains along the loading direction, which in turn reduces the entropy of the material. This first temporary shape can be fixed by ③ cooling to a temperature *T* such that *T*_trans,1_ > *T* > *T*_trans,2_, where the elastic energy is stored inside of the material thanks to the phase characterized by *T*_trans,1_. The second programming process can start by ④ deforming the material once again while the temperature is between both *T*_trans_ values, which further decreases the entropy of the material. The second temporary shape is then fixed by ⑤ cooling below *T*_trans,2_. Once again, the newly generated phase fixes the shape of the material and stores the extra elastic energy generated during step ④. If the freestanding material is ⑥ reheated above *T*_trans,2_, its shape will change from the second to the first temporary shape due to the release of the energy stored during step ⑤. ⑦ A further increase in the temperature above *T*_trans,1_ will reactivate the mobility of all polymer chains in the network, and the remaining stored energy will be released. This causes the shape recovery from the first temporary shape to the permanent shape.

In the last few years, several polymers have been reported to exhibit triple shape memory. Some examples of these materials based on a broad temperature interval can be found in the literature.^55−57^ On the other hand, references on triple shape memory materials based on two individual *T*_trans_ values are also available.^58−60^ Furthermore, the triple shape effect has also been observed in polymer laminates where each layer had a different *T*_trans_.^61^ A triple shape memory composite was created by Tobushi et al.^62^ that functions as a bending actuator. It is a laminate of a mix of SMP and shape memory alloy (SMA) that bends in two different directions depending on which *T*_trans_, that of the SMP or that of the SMA, was surpassed. As was already mentioned, conventional triple (or multiple) SMPs and SMCs are based on the 1W-SM effect,^63^ thus making the shape memory effect an irreversible feature requiring the application of the programming processes for further shape change. Nevertheless, a novel triple SMP exhibiting 2W-SM is reported.^64^

The same concept can be extended to multiple (quadruple, quintuple, ...) SMMs by chaining more than two programming processes, hence leading to more shape recoveries. In general, this is done in polymer networks that present very broad transition temperature intervals.^56,65,66^

A few references are available reporting the cyclic quality of SMMs during a high number (≥1000) of 1W-SM^67^ and 2W-SM^68^ cycles, still showing remarkable shape memory characteristics. However, we have not found reports regarding a high number of cycle repetitions involving multiple shapes. Some reports address the *R*_F_ and *R*_R_ of multiple shape memory materials for the first ≤6 cycles.^55,58,59,64,69^ Generally, it is observed that the *R*_F_ decreases and the *R*_R_ increases with each performed (sub)cycle. In comparing a triple SMM to a quadruple SMM, Dolog and Weiss reported a decrease of *R*_R_ and an increase of *R*_F_ when adding an extra shape to their cycle.^55^ More research is needed on the repetition of cycles and subcycles of multiple shape memory materials in order to assess the lifetime and quality of the behavior of these materials upon extended use.

### 2.3 Electro- and Magnetoactivation

Up to now, the notions and definitions given in this section are common to thermally triggered SMMs, regardless of the type of activation. In electroactivated thermally triggered SMMs, the temperature is a result of Joule resistive heating, which requires the flow of an electric current *I* through a material with a certain electrical resistance *R*. The power dissipated *P* is the product *P* = *RI*^2^ = *V*^2^/*R*, where *V* is the voltage drop across the material. From the formula of the dissipated power, it is straightforward that, at finite voltages and currents, *P* = 0 either for a perfectly conducting (*R* = 0) or perfectly insulating (*R* = *∞*) material. For resistive heating to take place, the material should ideally have a “medium” electrical resistance *R*, taking into account that reasonable values of currents (e.g., <100 mA) and voltages (e.g., <10 V) need to be used. In practice, reaching a dissipated power leading to a measurable temperature increase, e.g. *P* ≈ 0.1 W, requires resistance values usually in the rage of ∼100–10000 Ω, which is significantly lower than the resistance of the polymer. Such resistance values are achieved by incorporating electrically conductive fillers in the insulating SMP matrix.

Another alternative way of triggering the heating of the material and, thus, the activation and shape recovery of SMCs is by the application of alternating magnetic fields. In order to obtain magnetosensitive SMCs, ferromagnetic fillers need to be incorporated inside the SMP. There are three magnetic heating mechanisms that can arise in magnetosensitive SMCs: (i) eddy currents, (ii) hysteresis, and (iii) losses related to the rotation of the magnetic spin due to Néel–Brown relaxation.^70^ The heating efficiency is highly dependent on the size of the magnetic fillers. The composites with embedded fillers in the microscale can be heated through eddy current and hysteresis loss by applying magnetic fields at moderate frequencies. In contrast, when magnetic fillers in the nanoscale are introduced in the SMP, these two heating mechanisms become less effective. Hysteresis loss decreases and becomes negligible for particle diameters below a certain threshold. For instance, iron oxide (Fe_3_O_4_) turns superparamagnetic, i.e. the magnetization curve shows no hysteresis, for diameters smaller than 20 nm. Furthermore, a multidomain structure is theoretically estimated to happen for diameters higher than 62.9–128 nm.^71,72^ These characteristic sizes depend on the nature and shape of the magnetic nanoparticles.

The main magnetic heating mechanism for magnetic nanoparticles is related to Néel–Brown relaxations. High frequencies are needed for this type of magnetic heating to generate a considerable temperature increase. The Néel relaxation consists of the rotation of the nanoparticle magnetic moment to align with the direction of the applied magnetic field while the nanoparticle itself stays motionless. The Brown relaxation leads to the physical rotation of the magnetic nanoparticles. Both relaxation mechanisms exist simultaneously when the particles are subjected to AC magnetic fields. Nevertheless, the Néel relaxation tends to dominate for smaller nanoparticles (<10 nm in diameter) in viscous media and Brownian motion tends to dominate for larger particles in media with low viscosities.^70,73^

## 3 Electro- and Magnetoactive SMCs: Fillers and Activation

SMPs are preferred over SMAs for various applications due to their low cost and their ability to sustain large deformations. Nevertheless, they also present some drawbacks or limitations in comparison to SMAs: SMPs have lower strength, stiffness, and recovery force and are electrically and thermally insulating. In order to overcome these limitations, different fillers have been incorporated within SMPs: hence, the terminology shape memory polymer composite. The addition of some of these fillers has been proven to improve some properties and give extra characteristics to the pristine polymers themselves. This includes the possibility of different types of stimuli for triggering the necessary temperature difference in order to make use of the shape memory properties. The properties of the resulting SMCs can be affected by several factors such as filler distribution, filler–polymer interface, filler size and aspect ratio, the nature of the SMP, and even the processing techniques.^74^

**Figure 3:**
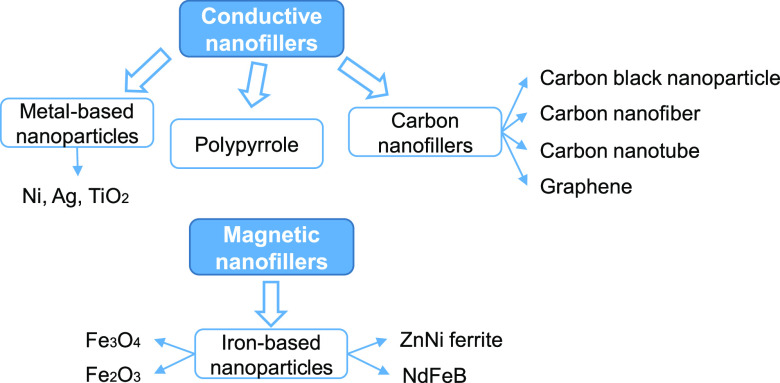
Main existing conductive and magnetic nanofillers reported in the literature to conform electro- and magnetoactive shape memory polymer composites.

Regarding the size of the fillers, continuous fibers such as carbon, glass, or aramid fibers have been used to reinforce SMPs.^75−77^ An alternative to continuous fibers is to use short (chopped) fibers^78,79^ of a few millimeters in length. SMA wires or fibers have also been reported in the literature.^80^ The fibers are generally incorporated within the SMP with the aim of improving the mechanical properties of the material. Even though not common, a few references have been found where carbon fibers, thanks to their high electrical conductivity, were also used to heat the SMC by means of electric Joule heating.^81−83^ This review focuses on shape memory composites with dispersed nanofillers that are used in order to give the resulting nanocomposite the ability to be heated with an electric current or a magnetic field. A schematic diagram showing an overview of the main existing nanofillers is shown in Figure 3.

### 3.1 Carbon Nanofillers

In the past couple of decades, shape memory polymer nanocomposites with embedded carbon nanofillers have been intensely investigated to electrically induce the shape memory effect. Thanks to their excellent electrical conductivity, these nanofillers facilitate resistive heating and permit the electrical activation of the shape memory characteristics of SMCs. Moreover, the thermal conductivity of the resulting composite is improved. This feature is particularly interesting for applications where direct heat cannot be applied in order to trigger the shape memory effects. Such applications include, for instance, biomedical devices or self-deployable structures.^84^ Within the group of carbon nanofillers, investigations focus on SMCs with embedded carbon black nanoparticles (CBs), carbon nanofibers (CNFs), single-walled and multiwalled carbon nanotubes (SWCNTs and MWCNTs, respectively), and graphene.

#### 3.1.1 Carbon Black Nanoparticles

CBs are effective fillers that can be used for the reinforcement of shape memory polymers. Due to their spherical geometry, a high concentration of CBs is needed in order to surpass the percolation threshold. At low concentrations, the addition of CBs does not create an electrically conductive network that has the ability to heat due to an electric current. Nevertheless, concentrations of 0.5–1.0 vol % CBs have been shown to improve the actuation ratio of the 2W-SM effect and the elastic modulus at high temperature of a polyethylene-based SMC.^39^ The same work showed that the 2W-SM effect of the composite was lost when the concentration of CB was increased to 20 vol %. At the required high concentrations for resistive heating at moderate voltages (>15 wt %), this family of SMCs was reported to have lower recovery ratios,^85^ more brittle behavior leading to failure of the material at shorter deformations,^86,87^ and, in some cases, severely worsened shape fixity.^88^ The last effect is found in semicrystalline SMP composites, since the addition of CBs decreases the amount of crystalline regions within the composite,^39^ which are in charge of fixing the temporary shape.

In order to achieve an electrical conducting network without a severe worsening of the shape memory characteristics of SMCs, other conductive fillers may be considered. Carbon nanotubes (CNTs) are an excellent alternative.

#### 3.1.2 Carbon Nanotubes

CNTs are conductive fillers with other advantageous characteristics that enable the formation of SMCs with low electrical resistivity at low filler concentrations. This is due to the high aspect ratio of CNTs, which facilitates the formation of an electrically conductive network within the composite. In other words, the percolation threshold for nanocomposites using CNTs is attained at lower concentrations than for other conductive fillers such as CBs.^89^ The resistivity once past this threshold for a given concentration of CNTs is also found to be lower than for other fillers at the same concentration. This can be advantageous, because a higher concentration of fillers may result in more brittle materials, high cost, high weight, or difficulty in processing due to an enormous increase in viscosity.

**Figure 4:**
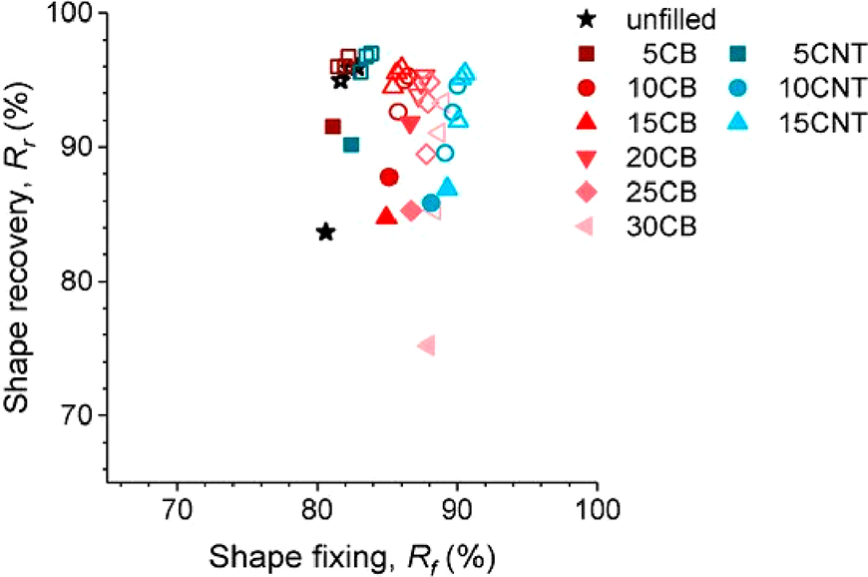
Relationship between the shape recovery ratio and the shape fixity ratio of a SMC with different concentrations (in phr) of CBs or CNTs (reproduced with permission from ref (87); published by MDPI, 2022.)

It also has been shown that, at the same concentrations, CNT-filled SMCs exhibit higher shape fixity than other fillers, such as CBs,^87^ which may be attributed to the alignment of the CNTs in the direction of the deformation. In the same work, the authors also showed that their shape memory elastomer filled with CNTs displays a better shape recovery ratio and better overall shape memory characteristics, as can be seen in Figure 4. The shape recovery was electrically triggered in their SMC with 15 parts per hundred rubber (phr) of CNTs at 50 V within 15 min. Conversely, the nature of the polymer matrix itself also influences the shape memory characteristics of the resulting composite. In a recent work, Tekay studied polymer blend networks of polycaprolactone (PCL) and a maleic anhydride grafted block copolymer (denominated SEBS-*g*-MA) with and without dispersed MWCNTs.^90^ The author showed that varying the composition of the polymer network toward a 50/50 blend increases the recovery and fixity ratios. Changing the relative concentration to 30/70 of PCL/SEBS-*g*-MA decreased these ratios by 35% and 3%, respectively. The SMC with 10 phr MWCNTs has a resistivity of 0.0382 Ω m that is able to undergo electrically triggered shape recovery (91.17%) when it is subjected to a constant voltage of 40 V in 56 s.

The method of dispersion of the CNTs also plays an important role in the electrical and mechanical properties of the SMCs: investigations have focused on how to avoid CNT aggregation due to van der Waals forces and achieve a random but uniform dispersion within the composite. It has been shown that mini twin-screw melt mixing,^91^ cross-linking MWCNTs onto semicrystalline networks,^92^*in situ* polymerization,^93^ or surface modification^94^ can decrease the electrical resistivity of the SMC. The last three methods also enhance the interfacial cohesiveness between the nanotubes and the polymer matrix, which is directly related to a better stress distribution and strength of the resulting composite. Nevertheless, surface modification should be used with caution since it may detrimentally affect the shape recovery ratio during the lifetime of the composite.^94^ Furthermore, a decrease in the electrical resistivity with an aggressive functionalization of CNTs is due to the generation of defects on the surface of the nanotubes.

Another solution for creating a conductive network with carbon nanotubes is by incorporating them in films or yarns onto the polymer. These are commonly referred to as MWCNT nanopapers or buckypapers, which have been shown, for example in the work of Lu and Gou,^95^ to facilitate the electrical activation of the shape memory composite when it is subjected to a constant current of 0.6 A or by Lu et al.^96^ to trigger the shape recovery of a composite at a constant voltage of 30 V while driving up a weight of 5 g by 30 mm. In another investigation, a shape memory epoxy matrix with MWCNT nanopaper was quickly cured by heating with an electric field at constant voltage, reaching 105 °C at 4.5 V.^97^ The electrical properties of the resulting composite were used once again to electrically trigger the shape recovery. A high concentration of 30 wt % MWCNTs could be embedded in the form of buckypaper in an epoxy shape memory polymer.^98^ This SMC has an elastic modulus 52% and 514% higher than the pristine material below and above the *T*_trans_, respectively. It shows shape recovery when it is subjected to 12 V under 22 s.

**Figure 5:**
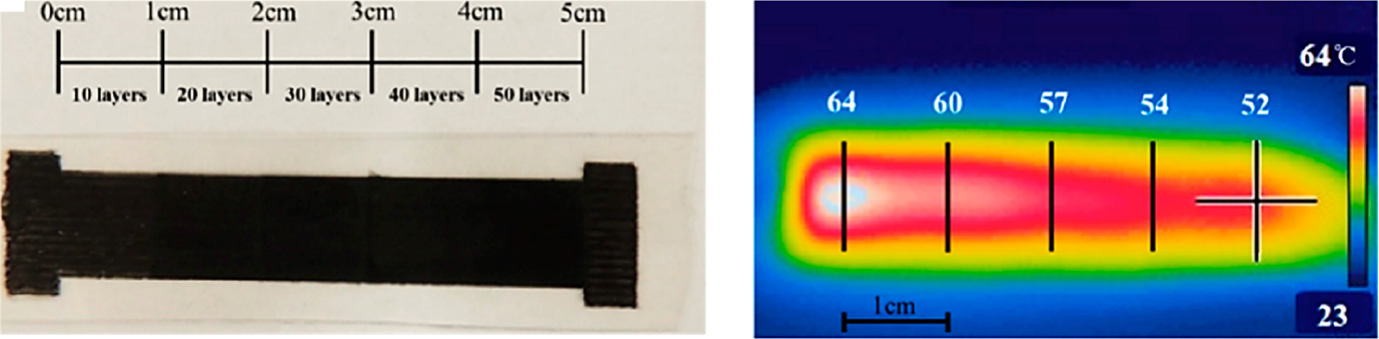
Stripe of a shape memory nanocomposite of polyurethane with different numbers of printed carbon nanotube layers along its length and the resulting temperature distribution due to resistive heating (reprinted from ref (99) with permission of Elsevier.)

Wang et al. reported on a novel way of fabricating CNT-filled polyurethane SMC by spraying-evaporation modeling.^99^ They printed CNT layers at different desired locations of the SMC. Several CNT layers can be printed in order to tailor the electrical resistivity of certain regions. More layers signify a lower electrical resistivity and higher temperature due to resistive heating whenever the material is subjected to a constant electrical current. They show a temperature difference of 12 °C along a single stripe of SMC between the regions with 10 and 50 CNT layers, as shown in Figure 5. This way, sequential and selectively triggered SMCs can be produced. They report complete shape recovery under 30 s when the SMCs are subjected to 40 V.

**Figure 6:**
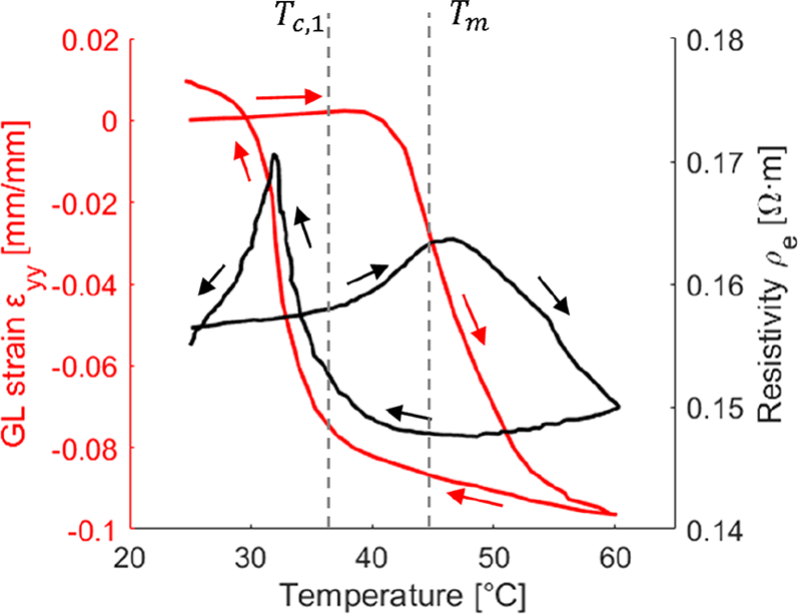
Evolution of the Green–Lagrange strain in the loading direction (ε_*yy*_ in red) and of the instantaneous electrical resistivity (ρ_e_ in black) with temperature of an SMP of polycaprolactone with 3 wt % MWCNTs. The test was performed at a constant stress of 600 kPa in order to study the 2W-SM cycle.

In the resistive heating phenomenon, the heat generated within the material directly depends on the injected electrical current and the electrical resistivity of the material. A thorough investigation on the phenomenon of resistive heating of an electroactive SMC was recently carried out by our group, including surface temperature measurements on a polycaprolactone SMP with 3 wt % MWCNTs used to validate analytical formulas and a 3D thermoelectric numerical model.^100^ This investigation shows that the electrical resistivity of the SMC has a nonlinear nonmonotonic dependency on temperature. Other material properties such as the heat capacity or the thermal conductivity are also shown to affect the resulting temperature of the SMC. We also used a bespoke tensile test bench with integrated controllers for resistive heating in order to investigate the evolution of the electrical resistivity of the same SMC during 1W-SM cycles performed with uniaxial tensile deformation.^101^ Using a proposed dedicated PI controller, the resulting temperature on the surface of the SMC due to resistive heating can be efficiently and accurately controlled in order to follow a certain heating/cooling ramp or in order to maintain a constant temperature even though the electrical resistivity varies with time. The results presented in ref (101) give also indications of the interplay among the electrical, thermal, and mechanical properties of the electroactive composite within shape memory cycles. An example of this interplay is shown in Figure 6, where the strain in the loading direction is shown as a function of a thermal cycle performed at a constant stress of 600 kPa in order to investigate the 2W-SM characteristics of the SMC. The electrical resistivity during the process is also depicted. Further information on these measurements can be found in ref (101).

**Figure 7:**
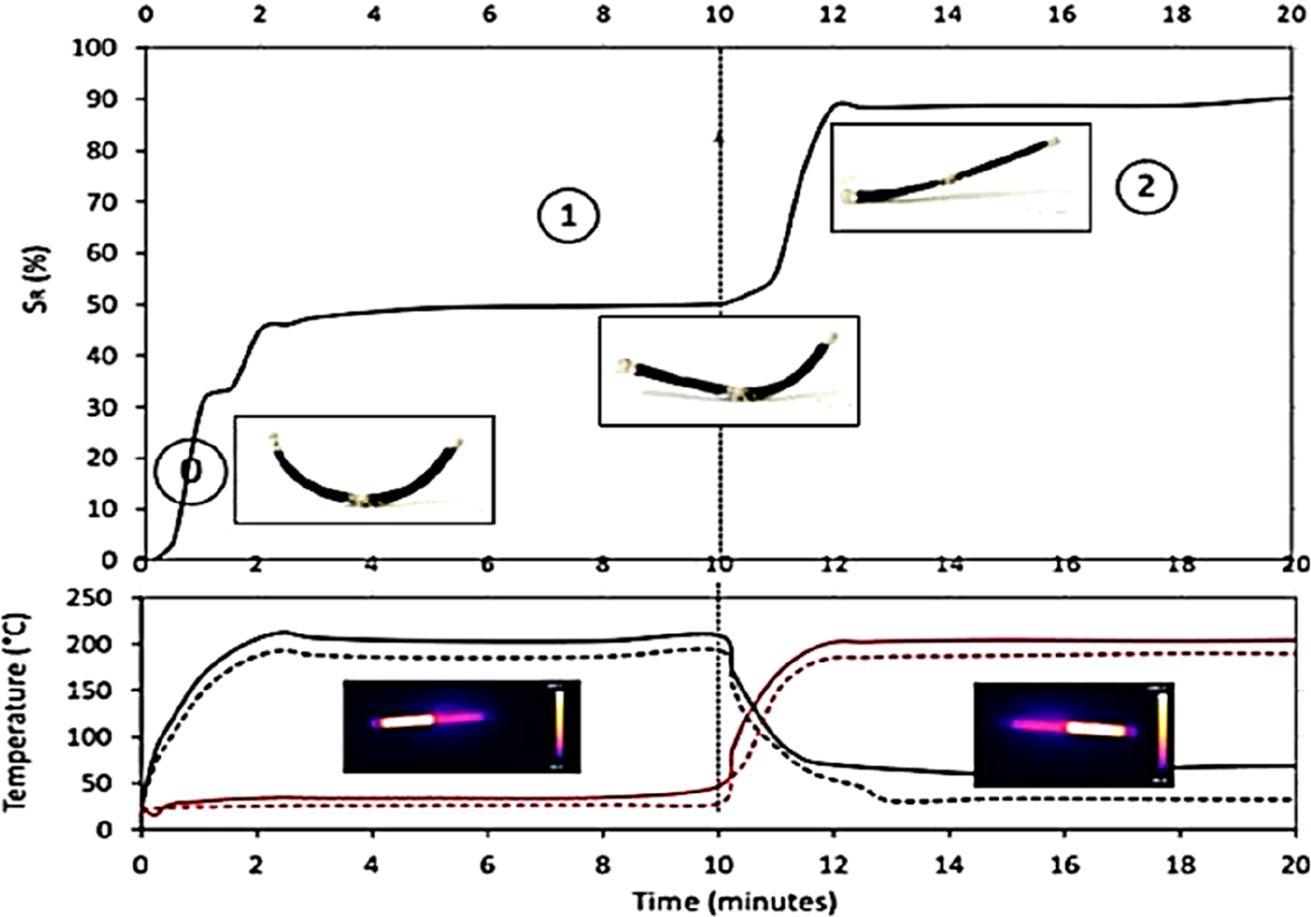
Sequential shape recovery due to selective resistive heating of a shape memory composite stripe. Three electrodes are painted on the surface of the SMC and are located on the right and left tips and another in between. The regions that heat up depend on which electrodes are used for injecting the electric current. During the first 10 min the current is injected between the central and left electrodes and during the last 10 min between the central and right electrodes (reprinted from ref (102), with permission of Elsevier.)

The energetic efficiency of the resistive heating process has been studied by Cortés et al.^102^ for a thermosetting epoxy SMP with 0.2 wt % MWCNTs. Furthermore, because the heating can be generated locally in the surfaces of interest, this last work also illustrates the ability of sequentially activating the shape recovery of different regions within a SMC plate, as shown in Figure 7, under a voltage in the range 126–265 V. The same research group studied the IR-activated shape recovery of printed shape memory acrylic resins with 0.1 wt % MWCNTs.^103^

SWCNTs have also been used within SMPs in order to achieve a multiresponse. In their work, Xiao et al. showed the beneficial characteristics of a pyrene-based SMP when 1–3 wt % SWCNTs was dispersed in a PCL copolymer in order to obtain a SMC that can be thermo-, electro-, or photoactivated.^104^ Upon the application of a constant voltage of 50 V, the SMC heated to 65 °C in 20 s, which proved satisfactory during shape recovery.

Electrically assisted CNT dispersion has been used in order to align CNTs within polymer matrices. Electric fields have successfully been applied during curing of polymer networks in order to successfully orient the conductive fillers in the direction of the current.^105^ Martin et al. showed that AC electrical fields achieve more linearly oriented conductive fillers in comparison to DC electric fields, which results in a more inhomogeneous and branched dispersion.^106^ These highly oriented nanocomposites overcome the percolation threshold at lower concentrations and show anisotropic electrical properties as well as decent optical transparency. Yu et al. created a SMC with embedded CB where the chained CNT dispersion was shown to decrease the electrical resistivity by over 1 order of magnitude in comparison to the SMC with a randomly oriented distribution.^107^

#### 3.1.3 Carbon Nanofiber

Carbon nanofibers (CNFs) have been reported as a cheaper alternative to CNTs to improve the electrical conductivity of a PCL shape memory polymer matrix.^108^ The resulting electrical resistivity for a concentration of 10 wt % is reported to be double that of the composites with CNTs at the same concentration. In an attempt to further decrease the electrical resistivity with carbon nanofibers, another PCL-based composite using carbon fiber felt (CFF) was reported by Gong et al. to have an electrical resistivity of 0.78 Ω m at a lower concentration of 3.6 wt % CFF.^109^

#### 3.1.4 Graphene

Graphene is another carbonaceous filler that is used in SMCs due to its excellent electrical and thermal conductivities. To facilitate a homogeneous dispersion within the SMP, graphene oxide (GO) and reduced graphene oxide (rGO) are usually utilized. Wang et al. investigated an epoxy nanocomposite incorporating rGO nanopaper that underwent complete shape recovery within 5 s by applying a low electric voltage of 6 V.^110^ The 2W-SM effect has also been shown in a shape memory polyurethane filled with graphene nanosheets.^111^ The work demonstrates the 2W-SM effect that can be electrically triggered for higher concentrations of 4 and 8 wt %, which resulted in SMC samples with electrical resistivities of 5.49 and 1.17 Ω m, respectively. During the conventional 1W-SM effect, this SMC shows improved shape memory characteristics, amounting to 93% shape fixity and 95% shape recovery at concentrations of 2 wt %. The investigation reported in this work also shows a better thermal stability of the resulting composite, with higher melting and crystallization temperatures after the addition of the conductive filler. A similar trend can be found in amorphous shape memory nanocomposites: an increased glass transition temperature is observed in amorphous shape memory nanocomposites by the addition of graphene^112^ with respect to the pristine polymer. After a certain concentration of graphene within the SMP, the glass transition temperature and the shape memory properties start to deteriorate due to poor dispersion and aggregation of the fillers.^113^ This investigation reported an optimized concentration of 1.5 phr of GO inside the polyurethane SMP. The same research group also examined the electroactive shape memory performance of the SMCs.^114^ The composites with 1.5 phr rGO could not be heated with their available setup due to a relatively high electrical resistivity on the order of 15 Ω m. However, the electrical resistivity of their SMC with 2.5 phr decreased to 0.4 Ω m. The values of the electrical resistivity reported here are calculated from the data presented in ref (114). Shape recovery of this SMC was obtained by applying 50 V and reaching a temperature of 64 °C after 90 s. A much faster electric shape recovery was reported in a poly(vinyl acetate) SMC with 4.5 wt % rGO.^115^ The resulting SMC has an electrical resistivity 0.037 Ω m. By applying 70 V, full shape recovery was demonstrated to happen within 2.5 s. Similarly, graphene-filled PCL has shown to achieve satisfactory electrical resistivities for slightly higher concentrations: i.e. 0.09 Ω m for 7 wt % reduced graphene oxide.^116^ Graphene–MWCNT hybrids have also been reported in a PCL matrix.^117,118^ Although the electrical resistivity using such hybrid conductive fillers may be somewhat smaller than those reported for MWCNT-filled SMCs, as the price of graphene (or SWCNTs) is much greater than that of MWCNTs for a limited improvement in the electrical characteristics, limits graphene competitiveness.^119^

Concerning the effect of graphene in the mechanical properties of the resulting SMPs, Chen et al. reported increases of 64% and 71% in the tensile strength and elastic modulus by adding 3 wt % of rGO in a shape memory epoxy matrix.^120^ It has been widely observed in the literature that the addition of high concentrations of graphene-based fillers increases the tensile strength of the resulting composite. The improved load-bearing capabilities usually come at the cost of worse shape memory characteristics due to the agglomeration of graphene at high concentrations. Interestingly, Zhang et al. proposed a method of functionalizing GO in order to produce a SMC that has both high load-bearing capabilities with an elastic modulus of 456.7 MPa and an excellent shape recovery of 100%.^121^

For further information on graphene-filled shape memory polymers, the reader can consult a recently written comprehensive review on this topic written by Kausar^122^ and the references therein.

### 3.2 Ferromagnetic Nanofillers

One of the most used magnetic fillers in SMCs is Fe_3_O_4_ because of its biocompatibility and nontoxicity, which enables their utilization for medical purposes. Other magnetic fillers that have been reported in the literature are particles of Fe_2_O_3_, Ni, NiZn, NdFeB, or NiMnGa. Examples of materials with these and other fillers can be found in a recent review written by van Vilsteren, Yarmand, and Ghodrat.^123^ Even though some SMCs contain nickel-based magnetic nanoparticles,^124−126^ no reference was found where these particles were used for the magnetic heating of the smart composites, with the exception of NiZn ferrite nanoparticles. For instance, Buckley et al. used 10 vol % NiZn ferrite nanoparticles dispersed in their SMP and achieved a temperature increase of 11 °C after 40 s in a 545 A m^–1^ magnetic field at 12.2 MHz.^127^ The fact that nickel nanoparticles are not or are rarely used in magnetoactive SMCs may be due to the lower heating efficiency of the polymer with Ni than with other nanoparticles such as iron or magnetite.^128^ Furthermore, the use of nickel nanoparticles may be limited in magnetic heating due to the complex synthesis,^129^ carcinogenic effects,^130^ and the unavoidable formation of oxide layers on the nickel nanoparticles, which is shown to detrimentally affect the magnetic heating ability.^131^ By adding polymer coatings to fight these drawbacks, the heating efficiency of nickel nanoparticles is dramatically decreased.^129^

Conventionally, magnetic particles are incorporated in the SMP matrix by physical blending. Due to van der Waals and magnetic forces, the unmodified magnetic particles tend to aggregate. This, together with the weak compatibility between the particles and the matrix, results in unsatisfactory mechanical and shape memory properties.^132^ In order to reduce the agglomeration of particles and improve the properties of the composite, several techniques have proven satisfactory, such as filler surface modification,^132^ chemical modification of the polymer matrix,^133^ or mechanical ultrasonication.^134^

The shape memory characteristics of magnetosensitive SMCs has been investigated in terms of the heating mechanism.^135^ It was found that the shape fixity ratio was higher in magnetosensitive SMCs when they were activated with magnetic heating rather than activated with conventional heating. The shape recovery ratio at a given magnetic field intensity, on the other hand, depends on the concentration of the magnetic fillers. It is higher for conventional heating when a low amount of magnetic fillers is dispersed. Nevertheless, after a certain threshold is attained, magnetic heating also results in a higher shape recovery ratio than conventional heating. Moreover, a few years later, the same research group investigated different parameters that have an influence on the temperature that can be reached with inductive heating on a biodegradable multiblock copolymer filled with Fe_3_O_4_.^136^ The magnetic field intensity *H* was varied between 7 and 30 kA m^–1^ (corresponding to μ_0_*H* = 8.8–37.7 mT, where μ_0_ is the magnetic permeability of free space) with frequencies between 253 and 732 kHz. They found that increasing the nanoparticle concentration and the magnetic field strength resulted in higher temperatures. They also studied the influence of the surrounding environment, leading to a lower steady-state temperature in distilled water than in air, and a considerably slower heating phenomenon when the material was submerged in a saline solution. Furthermore, the composition of the magnetic nanoparticles and their distribution within the polymer matrix were investigated. They found that, with a homogeneous distribution of 10 wt % of Fe_3_O_4_ nanoparticles, the mechanical properties of the composite were not dramatically different from those of the pristine SMP, while observing and improvement in its shape fixity by 7% and the shape recovery ratio by >2% on the third shape memory cycle.

**Figure 8:**
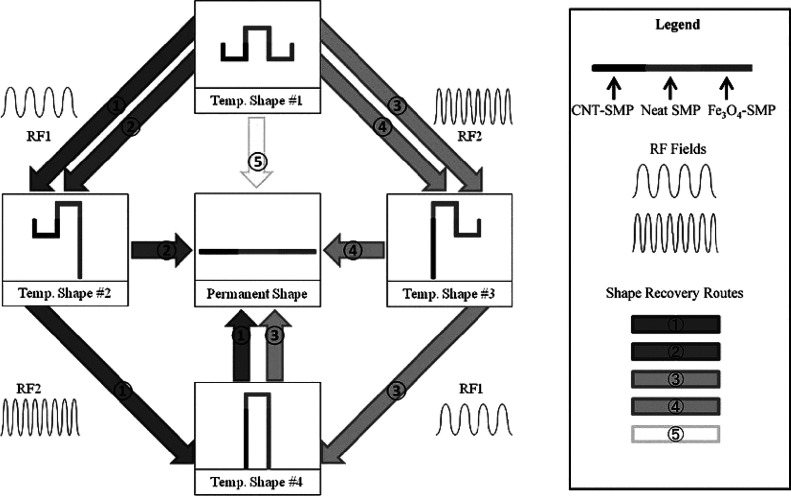
Illustration of the five shape recovery routes that can be followed from temporary Shape #1 to the permanent flat shape of a SMC with three different regions: CNT-SMP that can be activated with inductive heating at 13.56 MHz, neat SMP that can be activated with conventional heating, and Fe_3_O_4_-SMP that can be activated with inductive heating at 256 kHz. (Reproduced from ref (137) with permission of John Wiley and Sons. Copyright 2011 Wiley-VCH.)

An SMC with multiple fillers was fabricated by He et al.^137^ The conformation of the composite structure allowed for remotely and selectively triggering the shape recovery in specific regions in order to obtain different shapes. Their composite structure was divided into three regions with different compositions, as illustrated in Figure 8: on the right an epoxy SMP filled with 5 wt % Fe_3_O_4_ nanoparticles, on the center neat epoxy SMP, and on the left the epoxy SMP filled with 0.4 wt % CNTs. The selective triggering of the shape memory effect is due to a frequency effect: the region with Fe_3_O_4_ heats up by a magnetic field at 296 kHz, and the region with CNTs heats up by a magnetic field at 13.56 MHz. Among the plausible mechanisms responsible for the heating of the CNT-filled region when the material is subjected to a high-frequency magnetic field, one could be the presence of ferromagnetic metal impurities in the CNTs as a result of their manufacturing.^138−140^ After the shape programming process where the permanent (straight) shape is heated past the *T*_trans_ and deformed into the temporary shape (temporary shape #1), the recovery back toward the permanent shape can follow five different recovery routes with five total intermediate temporary shapes. Figure 8 illustrates each recovery path with a different arrow color (in gray values) together with a sketch of the required frequency of activation. The last step of each route toward the flat permanent shape needs to be achieved with conventional heating of the composite since the neat SMP central area does not contain any filler.

**Figure 9:**
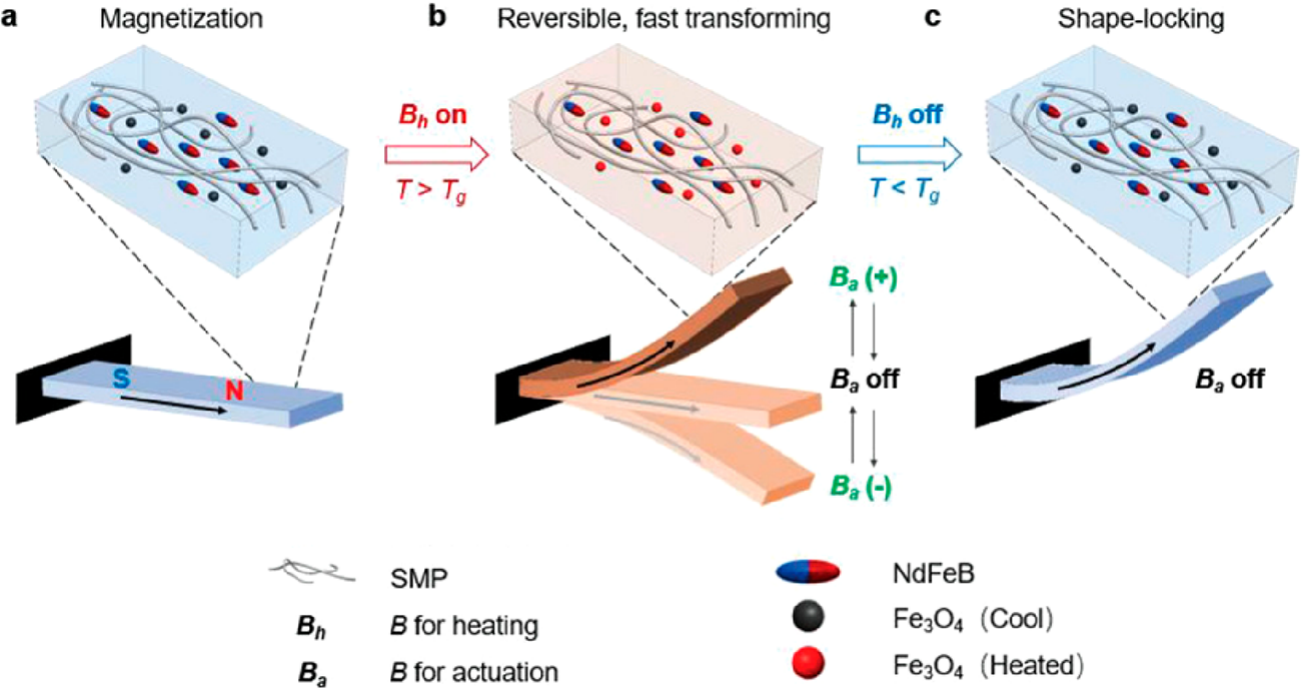
Principle of operation of a multishape magnetosensitive SMC with embedded NdFeB and Fe_3_O_4_ particles. (a) Illustration of magnetization and composition. (b) The SMC is heated by the application of a high-frequency AC magnetic field *B*_h_ above the glass transition of the SMP. Deformation and actuation can be achieved by a DC (or low-frequency AC) magnetic field *B*_a_. (c) Shape fixity can be achieved by removing *B*_h_ while keeping the desired *B*_a_. (Reproduced from ref (141), with permission of John Wiley and Sons. Copyright 2019 Wiley-VCH.)

SMCs with multiple ferromagnetic particles have also been produced in order to achieve a functional 2W-SM behavior.^141^ In this investigation, Fe_3_O_4_ and magnetized NdFeB particles were dispersed in an acrylate-based SMP. The Fe_3_O_4_ particles, with an average size of 30 μm, were heated by hysteresis loss with a high-frequency magnetic field of 10 mT at 60 kHz. The NdFeB particles, characterized by having huge coercivity, do not contribute to the heating due the small magnitude of the magnetic field. Instead, the NdFeB particles are used to generate a magnetic torque and induce a shape change when they are subjected to a DC magnetic field of 30 mT. As depicted in Figure 9, first the AC magnetic field *B*_h_ is applied onto the structure in order to heat up the material. When the *T*_trans_ is surpassed, the DC magnetic field *B*_a_ will cause the cantilever beam to bend. The new temporary shape can be fixed by removing the AC magnetic field while keeping the DC field. Otherwise, the material can be used as an actuator by keeping the AC magnetic field and varying the sign of the DC magnetic field at a low frequency (0.25 Hz), hence creating a reversible bending transformation of the beam. Besides the interesting potential applications and versatility of shape morphing and actuation of this material, the authors also proved the excellent shape memory characteristics, amounting to a shape fixity ratio of 95% and complete shape recovery.

Even though magnetic particles are normally embedded inside SMPs to enable magnetic heating of the resulting composite, some research groups have used them as means of increasing the electrical conductivity of the polymer matrix and heated the resulting composite with the Joule effect by injecting an electric current. Leng and co-workers have produced a polyurethane SMP with dispersed Ni powder. They produced aligned Ni chains by using a magnetic field during the curing of the SMP.^142^ The aided alignment of the magnetic nanoparticles helped to reduce the electric resistivity to <0.1 Ω m for 20 vol % of Ni powder. Resistive heating of up to 55 °C was shown when applying 6 V.

### 3.3 Other Fillers

Noble metals in the form of nanoparticles have attracted interest as fillers of SMPs in order to achieve multistimuli SMCs. This is due to their photothermal effect, where they absorb light at certain wavelengths from UV to near-IR and convert it into thermal energy. In their investigation, Mishra and Tracy created two similar SMCs filled with either gold nanospheres or gold nanorods, and they were able to selectively trigger the shape memory effect depending on the wavelength of light used: 530 nm for the nanospheres and 860 nm for the nanorods.^143^ Other noble metals that have been used to photothermally activate SMCs are platinum^144^ and silver nanoparticles.^145^ Besides nanoparticles of noble metals, metallic ions have been incorporated as fillers in SMCs to activate the shape memory effect using light in the near-IR. Bai et al. reported a composite made by cross-linking poly(acrylic acid) and poly(vinyl alcohol) with different metallic ions.^146^ They looked into Cu^2+^, Cd^2+^, Cr^2+^, Al^3+^, and ions of ferromagnetic materials (Fe^3+^, Co^2+^ and Ni^2+^).

The SMCs containing polypyrrole (Ppy) can take advantage of the electrical conductivity of the particles for electrically triggering the shape memory effect.^147,148^ Moreover, Ppy-SMP composites are also shown to exhibit an excellent photothermal performance. Thus, these SMCs are multistimuli. There are many other fillers that have been used in shape memory polymer composites in order to improve mechanical, thermal, and electrical properties or to obtain multistimuli materials. For example, shape memory alloys have been embedded in SMPs in the form of wires^149^ or particules.^80^ Silicon carbide has been used due to its temperature stability and good microwave absorption that allows the SMM to be activated through multiple stimuli.^150^ Oxides of tungsten, silicon, or aluminum have been incorporated in order to improve the mechanical properties of SMC foams for medical devices.^151^ Other electroactive polymers have been blended with SMPs,^152^ liquid metals have been used to fill the SMP and achieve electroactivation,^153^ and fillers such as cellulose nanocrystals^154^ or nanoparticules of mineral silicates^151^ have been used to reinforce the SMP but do not enable electro- or magnetoactivation.

After this overview of fillers in SMCs, the reader can proceed to the Appendix, which lists some of the many existing references on electro- and magnetosensitive SMCs, together with the activation mechanism and general characteristics of the materials reported.

## 4 Applications

In view of the highly tailorable material properties of SMCs together with the excellent shape memory properties and possible and versatile multistimuli activation, SMCs are used (or intend to be used) in many areas of research and industry. In this section, a few applications of shape memory composites are described, paying special attention to those that can be electrically activated.

### 4.1 Biomedical Industry

**Figure 10:**
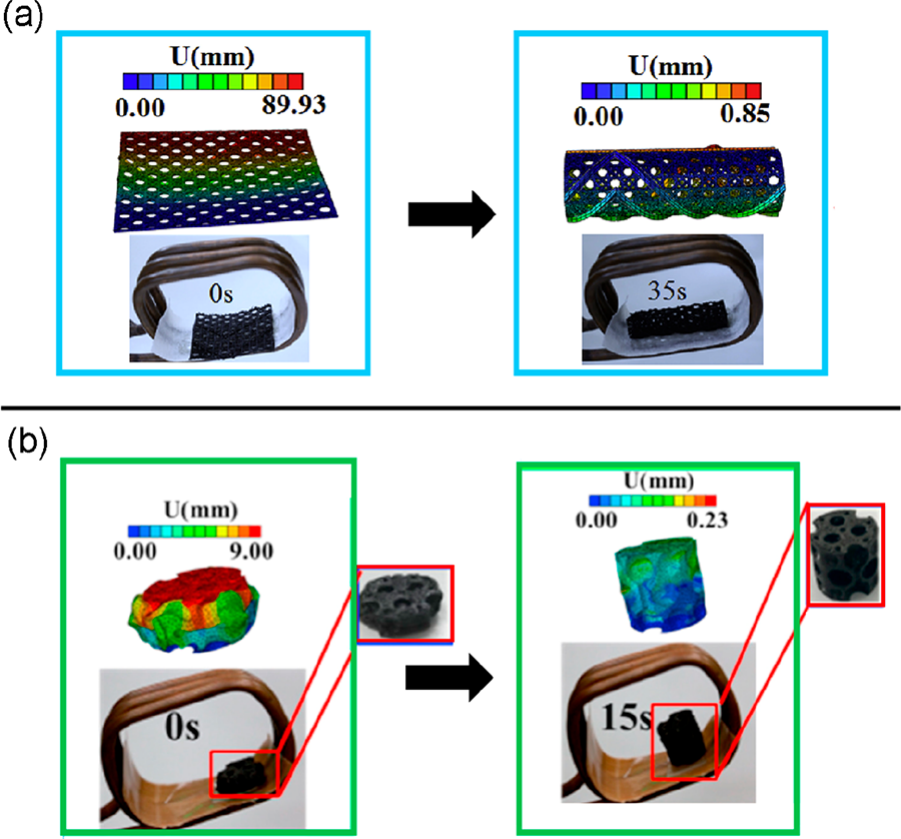
4D printed poly(lactic acid) SMP with embedded Fe_3_O_4_ composites actuated by a magnetic field for the conception of (a) tracheal scaffolds (reprinted from ref (156), with permission from Elsevier) and (b) porous bone tissue scaffolds (reprinted from ref (158). with permission from Elsevier.)

Within the biomedical industry, shape memory polymers have attracted a great deal of attention. Most of the applications in this field use conventional heating of SMPs^4^ or heating with magnetic fields of SMCs with magnetic nanofillers. A composite made from a poly(lactic acid) SMP with dispersed 10 wt % Fe_3_O_4_ nanoparticles was used in 4D printing in order to fabricate prototypes of shape memory heart occluders for the treatment of congenital heart diseases.^155^ A similar SMC was used for conceiving a tracheal scaffold.^156,157^ The composite, with 15 wt % Fe_3_O_4_, is to be implanted as a flat surface and, under the influence of an AC magnetic field of 4 kA m^–1^ at 30 kHz, it curls to form a tube within 35 s, as shown in Figure 10a. Moreover, the same research group also reported on the feasibility of this SMC to create different designs of bioinspired scaffolds to be applied for bone repair and regeneration.^158^ One of the proposed structural designs is shown in Figure 10b, where the magnetic activation (4 kA m^–1^ at 30 kHz) expands the SMC scaffolds within 15 s. As an additional example, magnetoactive SMCs have also been conceived in the biomedical field to apply in drug delivery^159^ or as intravascular stents.^160,161^

**Figure 11:**
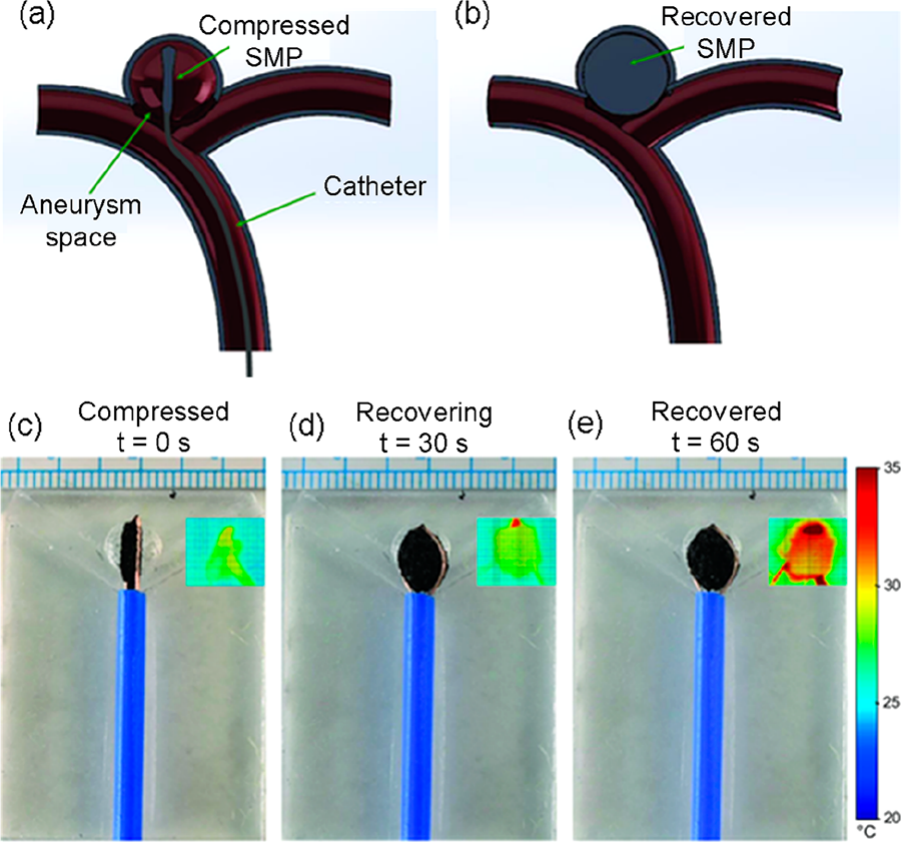
Illustration of the treatment of aneurysms by filling them with a SMC. (a) Compressed SMC foam being placed in a saccular aneurysm via a catheter. (b) Recovered expanded shape of the SMC filling the aneurysm space. Demonstration of the prototype for the treatment of aneurysms of a SMC with 1 wt % CNTs at (c) 0 s, (d) 30 s, and (e) 60 s after the injection of an electric current (1 A). (Reproduced from ref (162) with permission of John Wiley and Sons. Copyright 2021 Wiley-VCH).

Electroactive SMCs have also been found to be the material of choice for biomedical applications in the literature. For example, a porous SMP foam with homogeneously dispersed CNTs has been fabricated for the potential treatment of intracranial aneurysms by implantation through a catheter (Figure 11a) and subsequent expansion of the foam in order to fill the aneurysm, as illustrated in Figure 11b.^162^ Thanks to the incorporation of the CNTs, X-ray imaging can be used during the implantation procedure to visualize the path of the SMP foam. The researchers used a foam with 1 wt % CNTs and investigated the steady-state temperature reached due to the injection of a constant current. After compressing the foam to 60% of its initial volume, the authors triggered shape recovery to the expanded foam in less than 1 min by injecting 1 A (Figure 11c–e), demonstrating in this way the potential of the SMC in the treatment of aneurysms. A similar investigation was reported in their previous work with 0.005 g mol^–1^ CNTs.^163^ The applied deformation toward the temporary shape is a compression of more than 50%. Complete electrically triggered shape recovery toward the expanded permanent shape happened within 2 min at 0.2 A and reached a maximum temperature of 46.8 °C, preventing neighboring tissue damage.

### 4.2 Aerospace Industry

**Figure 12:**
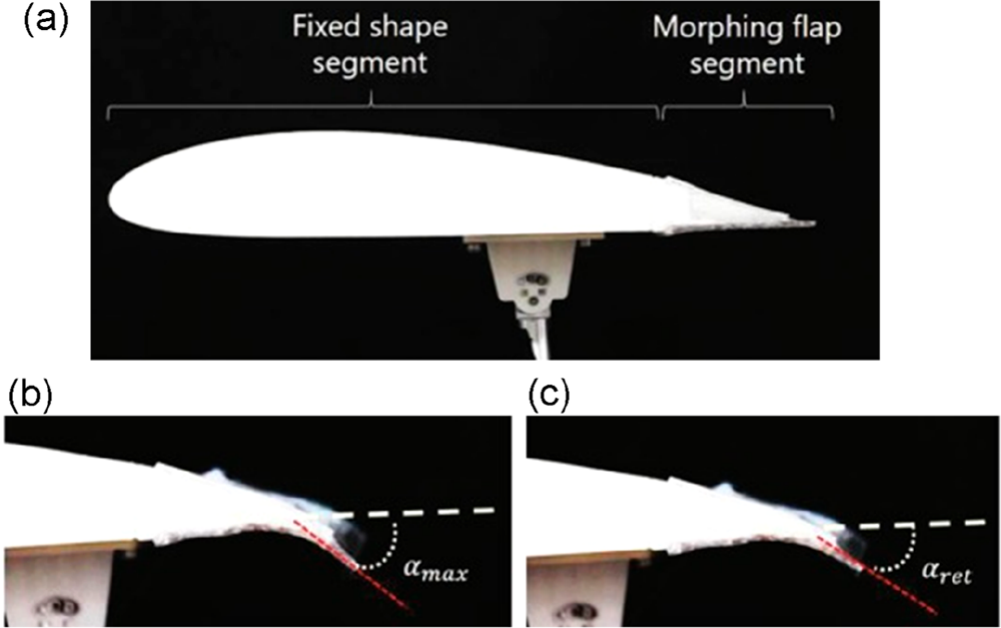
(a) Wing of an unmanned aerial vehicle with a morphing flap at the trailing edge capable of changing the deformation angle between (b) 39° and (c) 32°. The actuation mechanism is through resistive heating of both the SMA wires and the SMP–nichrome composite. (Reprinted from ref (165), with permission from Elsevier.)

Within the aerospace field, self-deployable structures have been reported using magnetoactive SMCs. A reconfigurable morphing antenna was reported to be heated in an AC magnetic field of 40 mT at 60 kHz to be used as a self-deployable structure or as a structure with variable resonant frequencies.^141^ Regarding electroactive SMCs, Paik et al. fabricated a SMC made of polyurethane with 7 wt % MWCNTs by *in situ* polymerization. The electrical resistivity of the SMC was around 0.8 Ω m. The SMC was applied in a microaerial vehicle to actuate the control surface (rudder).^164^ When an electric current was injected in the SMC, it shrunk and produced a 30° angle deflection. Unfortunately, because this was a 1W-SM effect, subsequent shape memory programming was be needed for further deflection of the control surface, which is not compatible with realistic flight control. More recently, a hybrid shape memory composite actuator has been developed as a laminate composed of SMA wires and SMP filled with nichrome for morphing flap flight.^165^ Resistive heating was induced in the composite structure by injecting a current of 0.7 A in the SMA wires and of 0.4 A in the SMP–nichrome composite. The authors used this composite for the morphing of the trailing edge of the wings of an unmanned aerial vehicle, resulting in deformation angles between 32 and 39°, as shown in Figure 12. The flapping of the trailing edge proved to create an extra lift force on the wing while decreasing the drag.

### 4.3 Soft Robotics

Using a magnetoactive SMC made of poly(aryl ether ketone) with dispersed Fe_3_O_4_ nanoparticles, Yang et al. showed the diversity of potential applications of their composite exhibiting a remotely and magnetically triggered 1W-SM effect.^166^ Their layered-film nanocomposite with 15 wt % magnetic nanoparticles was subjected to an AC magnetic field of 27.9 kHz for 50 s. They fabricated a ball launcher that propelled a ball forward by triggering the shape recovery with the AC magnetic field. Moreover, they showed the morphing abilities of their composite material with flower-shaped and plane-shaped geometries that unfolded back to a flat position with the application of the magnetic field. Other soft robotic applications were envisioned by, for instance, Cohn et al., using their SMC of polycaprolactone dimethacrylate with 5 wt % of Fe_3_O_4_ nanoparticles.^167^ In their published work, they showed the morphing of several structures when the material was subjected to a magnetic field (4 kA m^–1^ at 375 kHz) such as a self-deployable honeycomb cylinder or a spider web and the folding of a spiral. They investigated rolling and gripping movements using their SMC.

**Figure 13:**
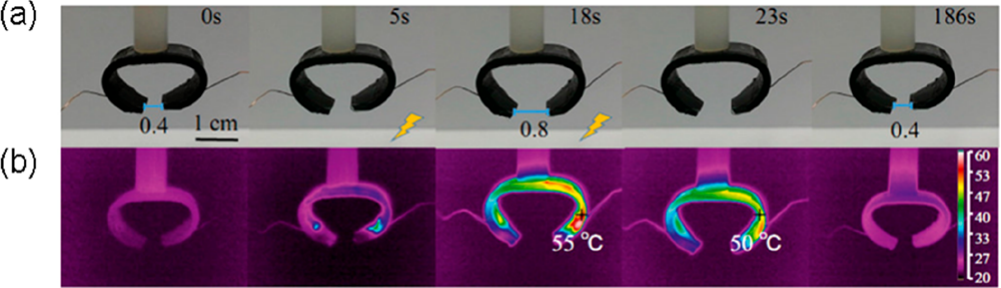
(a) Photographs and (b) IR thermal images of the SMC electroactive gripper. (Adapted with permission from ref (48). Copyright 2019 American Chemical Society.)

A crawling robot was reported by Peng et al.^168^ In this work, they used a double layer of a 3D porous CNT sponge SMP to create a nanocomposite that was able to undergo a 2W-SM effect when it was subjected to resistive heating. The CNT loading inside the composite was of 1.08 wt %, which resulted in an electrical resistivity of about 0.007 Ω m. They recreated an inchworm locomotion by applying voltages between 2 and 8 V. The inchworm-like robot is able to travel 1.2 cm in 10 min. Xu et al. produced another SMC made of poly(ethylene-*co*-octene) filled with CNTs to achieve a composite was is able to undergo a stress-free 2W-SM effect with a fast response when it was subjected to either low voltages (<36 V) or infrared light (250 mW cm^–2^).^48^ In this work, they built an electroactive gripper and a photoactive crawling robot. The gripper, shown in Figure 13, was formed in a C shape. At room temperature, the opening of both tips of the C shape was 0.4 cm. Upon the application of 36 V, the temperature of the composite increased to 55 °C and the shape changed by increasing the aperture to 0.8 cm in 18 s. When the material was cooled once again, the original opening of 0.4 cm was recovered after 168 s. They showed the lifting capabilities of this gripper by lifting 15 nuts (69.09 g). In their most recent work, Xu et al. also reported a high-temperature warning robot that was made possible by the addition of paraffin wax into the composite.^169^ They hot-pressed the SMC layer with sandpaper to make the surface uneven, and they stacked two uneven-surfaced layers together. At high temperature, the stiffness of the SMC decreased and the surface roughness disappeared, leaning to a better contact between both layers that could be monitored thanks to a change of electrical resistivity across the layers of more than 2 orders of magnitude.

Other works have also exploited the weight-lifting and 2W-SM and multiple shape capabilities of SMCs. Xu et al. used a poly(ethylene-*co*-vinyl acetate) filled with CNTs that was activated with a voltage.^170^ Instead of using the SMC as the grip, they mounted it in a setup that permitted the opening and closing of a mechanical grip due to the elongation and contraction of the SMC during its 2W-SM effect. Furthermore, Hu et al. built a device with a magnetoresistive triple SMC that was capable of displaying text in Braille and self-refresh in order to potentially apply it as a Braille e-book.^171^

### 4.4 4D Printing

Within 4D printing, Dong et al. investigated several applications in poly(lactic acid) filled with 8 wt % CNTs.^172^ They demonstrated the fast shape recovery (<44 s) of pyramid-, diamond-, and crown-shaped printed structures when they were subjected to voltages in the range 20–35 V. Interestingly, they also developed a multisegment self-deployable rectangular structure that can be selectively deployed in three sections. Each section was actuated with 25 V in order to reach a temperature >70 °C. Moreover, they also developed an external stent that can be implanted in an extended unfolded shape outside of the body part. By applying 20 V, the stent shrinks and bends within 60 s.

### 4.5 Self-Healing

Self-healing of structures is another popular application that has been investigated in the scope of electrically and magnetically triggered shape memory composites. This property can be used in a plethora of applications in order to self-repair surfaces and structures that have suffered from impact or fatigue, thus prolonging the life of the structure and delaying disposal. An example of this kind of composite was reported by Ren et al.^173^ It consisted of a polycaprolactone/thermoplastic polyurethane SMP with 2–6 wt % CNTs that exhibited fixity and shape recovery ratios of 96% and 94%, respectively. They reported excellent electrically triggered healing efficiencies of the composites ranging from 94.79% to 96.15%. Furthermore, comprehensive reviews on electroactive^174^ and magnetoactive^175^ self-healable nanocomposites have been recently published, where other examples of shape memory self-healable materials can be found.

Besides the applications that have already been reported in the literature, the interesting properties of electro- and magnetoactive SMCs make these materials excellent candidates for other prospective applications such as artificial muscles, self-deploying antennae and solar arrays, dental fixtures, self-foldable devices, and many more.^176,177^

## 5 Conclusions and Future Horizons

Bearing in mind all the information and the numerous references cited in this review, it is not surprising to say that SMPs and SMCs have attracted a great deal of attention and interest from researchers and the industry. Even though shape memory materials have been investigated for years, there are probably many things to discover, many properties to keep enhancing, and many applications to be conceived.

Considering the extensive literature review performed, some prospective future directions in the research field of shape memory polymer and their composites are listed here below.

(i) High importance has been given in recent research to minimize the time during shape recovery of SMCs for their application as actuators. This trend will likely continue in order to obtain faster SMC actuators. Besides the ability to achieve a fast recovery time, a variable time scale or even a fast interruption of the shape recovery may be desirable for some applications, notably in the biomedical field.
(ii) Materials with a tunable transition temperature are highly desirable, but further investigation is needed in order to do so accurately and repetitively. Furthermore, broad transitions may lead to multiple intermediate temporary shapes during a slow shape recovery. The accurate characterization, description, and prediction of these intermediate shapes could widen the application of these materials.
(iii) Due to the specific requirements of the biomedical industry, surely more investigations will follow on biocompatible SMPs and SMCs that can be remotely actuated. Besides, if they are also biodegradable, some implants would not need a secondary surgical intervention to be removed.
(iv) On the same green trend as biodegradability, dynamic covalent bonds on the SMP matrices will surely be kept under investigation. These allow for recyclability and reprocessability of the material. Similarly, research on self-healable SMCs is on the rise so as to increase the lifetime of the structures.
(v) Especially within the area of electrically activated SMCs, there has been recent interest in selectively and sequentially triggering the shape recovery. By improving the localization of heat generation, sequential shape recovery may lead to more applications of electroactive SMCs in self-deployable and self-foldable devices.
(vi) There are some cases where direct heating cannot be applied in order to trigger the shape recovery, such as when the material is surrounded by sensitive equipment or when it is inside of the human body. In general, the current trend in the investigation of SMCs is in multistimuli materials where, besides a conventional thermal treatment, alternative heating can be applied in order to trigger the shape memory effects remotely. These mechanisms include resistive heating, electromagnetic induction, and photothermal effects. An in-depth understanding of the energy conversion into heat of these alternative heating mechanisms may allow for complex and accurately actuated devices.
(vii) Because of the direct link between the material and the mechanical and shape memory properties of SMCs, a predictable and controllable temperature through alternative heating mechanisms (resistive Joule heating, electromagnetic induction, etc.) is desired.
(viii) Due to the one-time actuation or the required time-consuming shape reprogramming of conventional 1W-SM polymers and composites, materials exhibiting 2W-SM effects will likely be the object of future investigations in order to meet the challenging needs of future applications. Undoubtedly, more research is needed in this area to evaluate the fatigue and wearing of the 2W-SM characteristics in order to estimate the lifetime expectancy of these materials when in use. Similarly, the cyclic (or subcyclic) lifetime of multiple shape memory composites should be further investigated.

The outstanding and polyvalent characteristics of these materials offer an incredible platform to investigate and create applications that are, as of now, unimaginable.

## Appendix

This appendix provides Table 1, giving some of the many references available in the literature of shape memory composites with certain fillers that allows them to be activated with either an electric field or a magnetic field. Some other characteristics of the materials or the experiments are given as additional information. For a better understanding of the information listed in the table, please refer to the list of abbreviations given below.

**Table 1:** Compilation of References of Shape Memory Composites Activated with an Electric or Magnetic Field

SMP	filler	ς	ρ_e_ (Ω m)	activation	activation magnitude	*T* reached (°C)	*t*_R_ (s)	comments	ref
PCL	MWCNT	3 wt %	0.15–0.6	electrical	controlled resistive heating (0.6–15.7 mA)	60	130	1W-SM and 2W-SM effects; *R*_F_ > 96.7% and *R*_R_ > 88.9%; variable electric current to achieve constant temperature and constant heating and cooling rates; nonlinear evolution of ρ_e_ within shape memory cycle	(101)
PU	MWCNT + Fe_3_O_4_	30 wt % Fe_3_O_4_ +0.25–1 wt % MWCNT		magnetic	29.7 kA/m at 45 kHz	>45	60	*R*_R_ >95%	(178)
PU/SM-PU	CB	1–5 wt %	5 × 10^–2^ Ω m for 5 wt %; 10^–3^ Ω m for 3 wt %	electrical	100 V		25	2W-SM; *R*_*R*_ 99.8%	(179)
PU	MWCNT	5 wt %	∼1 Ω m	electrical	60 V	100	10		(180)
EP	MWCNT	0.2 wt %	1–10 Ω m	electrical	126–265 V	200–250	<300	sequential recovery	(102)
EP	MWCNT	30 wt %	47 Ω m	electrical	12 V	>55	22		(98)
EP	rGO	0.5 wt %	0.025 Ω m	electrical	10 V	>100	10	*R*_F_ = 95% and *R*_R_ = 98%; electrical recovery	(181)
PLA/PU	MWCNT and CF	6 wt %		electrical	10 V	>70	25	*R*_R_ = 94%; negative Poisson ratio	(182)
PVAl	MWCNT	5–30 wt %	0.52 Ω m for 30 wt %	electrical	45–120 V	>74	35		(183)
PCL	MWCNT coated Fe_3_O_4_			magnetic	6.8 kA/m at 20 kHz	*T* increase of 17.2	120	alignment of Fe_3_O_4_-coated MWCNT due to magnetic field 0.2 T; increase of elastic modulus by almost 100% but reduction of max elongation	(184)
PCL	MWCNT	3 wt %	0.05 Ω m	electrical	0.1 W	∼50		analytical, numerical, and experimental investigation	(100)
BR	MWCNT and CB	0–15 phr MWCNT or 0–30 phr CB	10^–2^ Ω m for 15 phr CNT or 30 phr CB	electrical	50 V	110 after 15 min at 50 V	900	lower ρ_e_ and higher *R*_F_ and *R*_R_ for CNT than CB	(87)
PU	Fe_3_O_4_	0–9 wt %		magnetic	250 A/m at 20 kHz	>52	35	*R*_R_ decrease with ς, slight *R*_F_ increase with ς	(185)
PBS–PEG	CNT	0.2–1 wt %	7.05 Ω m for 1 wt %	electrical	20–120 V	85 at 70 V for 1 wt %	55	CNT reduced ductility	(186)
PU	CNF	1–7 wt %	560 Ω m for 7 wt %	electrical	100–300 V	25–55		χ_c_, *R*_F_ decrease with ς of CNF; elastic modulus increase with CNF ς	(187)
PU	CNT	3 wt %	0.037 Ω m	electrical	50 V	>40	40	different preparation techniques and effect of dispersion and electrical properties	(93)
PU	G sheet	1–8 wt %	1.17 Ω m for 8 wt %	electrical	65–80 V	>70	8	2W-SM; study mechanical properties; *R*_F_ = 95%; *R*_R_ = 93%	(111)
PU	CNT	4 wt %		electrical	40 V	100 cross-linked CNT	50	CNT covalently bonded to PU; increase *R*_R_ and *R*_F_ for cross-linked CNT (>10%)	(92)
PU	rGO	0–2.5 phr	0.4 Ω m for 2.5 phr	electrical	50 V	64 for 2.5 phr	120	*T*_g_ and mechanical properties increase with ς; ductility increase with cycles; *R*_*R*_ decreases with ς	(114)
EOC	CB	0–38 wt %	0.08 Ω m for 17 wt %	electrical	5–15 V for 17 wt %	40–160	60	two different CB fillers; lower *R*_*R*_ for higher ς	(85)
PU	rGO + Fe_3_O_4_ and γ-Fe_2_O_3_	3 wt % GO + 7 wt % Fe_3_O_4_		magnetic	287 kHz with 300A in coil	70 for mixture, 60 for hybrid	60	Young modulus doubled from hybrid to only Fe_3_O_4_	(188)
PU	Ni powder chains	0–20 vol%	0.005–0.01 Ω m for 20%, 10^4^ for 4%	electrical	6 V	55	90	alignment magnetic field; decrease *T*_g_ with ς	(142)
PU	CB with and without 0.5 vol% Ni	4–10% CB	0.1 Ω m for 10% CB + chained 0.5% Ni	electrical	30 V	80	120	ρ_e_ increases with number of cycles	(189)
PVAl	CNT and G	20 phr GO and 4–16 phr CNT	0.1 Ω m for 20 GO + 16 CNT	electrical	60 V	100 max (normally 60)	120	ρ_e_ decreases with bending angle	(190)
PS	CNT	1.47–7.02 wt %	0.01 Ω m for 7.02 wt %	electrical	35 V	>85	80	*R*_R_ ≈ 100%	(96)
PU	GNP	1–3%	4 Ω m for 3% GNP	electrical	75 V	100 for 3%	60	χ_c_, elastic modulus, *R*_F_ and *R*_R_ increase with ς; strength, *T*_g_, and *T*_m_ decrease with ς	(191)
EP	Ag decorated rGO and CF mat	2–4 wt % rGO and 11.6% CF	2.3 × 10^3^ Ω m	electrical	8.6 V	100	36	*R*_R_ = 99%	(192)
PS	MWCNT nanopaper	1.47–7.02 wt %	0.008 Ω m for 7.02 wt %	electrical	0.6 A	>62	300	*R*_R_ = 98%	(95)
EP	CNF	9.18 wt %?	0.03 Ω m	electrical	10–20 V	>50	2.1	increase elastic modulus above *T*_g_, increase κ	(193)
PU	MWCNT	3–7 wt %	12 Ω m for 3 wt %	electrical	40 V	>70 in 10 s	9	Young modulus increase by 50%; *R*_R_ > 98%; *T*_c_ increase with ς	(194)
PU	Fe_3_O_4_	0–10 wt %		magnetic	30 kA/m at 258 kHz	70 at 150 s 12.5 kA/m 258 kHz	22	magnetic heating depends on geometry; for high ς, *R*_F_ and *R*_R_ higher for magnetic actuation	(135)
PK BMI	MWCNT	8 wt %	∼ 0.06 Ω m	electrical	20–50 V	40–140		*R*_R_ = 90% by 40 V; self-healing	(195)
PU	MWCNT	3–10%	0.9 Ω m	electrical	9.5 mA	32			(164)
PU	GO	0–20 phr	>7 × 10^2^ Ω m	electrical	100–200 V	<60			(196)
EP	Fe_3_O_4_ coated with oelic acid	1.5–8 wt %		magnetic	30 mT at 293 kHz	*T* increase of +25	60		(133)
PPC/PLA	MWCNT	1–3 phr	0.1 Ω m for 3 phr	electrical	20–30 V	130 max for PPC/PLA (50/50) 3 phr at 30 V	30	max tensile strength and strain at break increased with ς at room temperature; At 50 °C strength decrease with concentration; *R*_R_ = 97%	(197)
PLA/PU (70/30)	CB	0–8 phr	∼0.4 Ω m for 8 phr	electrical	30 V	98 for 8 phr	80	*R*_F_ = 90%; *R*_R_ increase from 59% to 85.9% for 8 phr CB; conductivity ∼1 order of magnitude higher for PLA/PU(70/30) than for PLA	(198)
PU	CF/PU yarn fabric	10.9% CF	1 × 10^–3^ Ω m	electrical	6 V	87	80	*R*_R_ = 99.3%; applications of deployable structure	(82)
PEVA	CNF	0–15 wt %	0.204 Ω m for 15 wt %; 5 × 10^9^ Ω m for 3 wt %	electrical	60 V	100 after 25 s		2W-SM; *R*_R_ decreases with ς	(199)
PU/PCL	modified MWCNT	2–10 wt %	1 Ω m for 10 wt %; 10^2^ Ω m for 4 wt %	electrical	40 V	>50	15	*T*_g_ of PLA increase with ς; *T*_g_of PU decrease with ς; *T*_c_decrease with ς	(200)
PU/PVDF (90/10)	modified MWCNT	0–10 wt %	50 Ω m for 10 wt % pristine CNT; 4 Ω m for 10 wt % modified CNT	electrical	40 V	*T* rise of +40	15	κ × 4 by adding 10 wt % MWCNT; increase 10% tensile strength and decrease 100% elongation at break for surface-modified MWCNT than pristine; *R*_R_ decrease with cycles faster for modified than pristine CNT	(94)
PU	Fe_3_O_4_	0–40 vol%	10^6^ Ω m for 40 vol%	magnetic	4.4 kA/m at 50 Hz	50	1200	electric percolation at 30 wt %; *C*_p_ decrease with ς; κ increased by 0.4 for 40 vol %	(201)
PEVA and PU	FE_2_O_3_	0–13.6 wt %		magnetic	15–23 kA/m	100 for PEVA; 108 for PU			(202)
PDL	Fe_3_O_4_	5–11 wt %		magnetic	7–30 kA/m at 258 kHz	100	180	decreased *R*_F_ (95% to 90%) and increased *R*_R_ (95% to 97%) for increased ς	(203)
EP	CB	0–10 wt %	200 Ω m for 10 wt %	electrical	3.5 W constant power	65	<200	ρ_e_ vs ε during elongation and recovery	(204)
PVA	GNP	1.5–4.5 wt %	0.04 Ω m for 4.5 wt %	electrical	50–70 V		2.5	elastic modulus increases by 1 order of magnitude from 0 to 4.5 wt %; *T*_g_ decrease with ς	(115)
PU	PPy	8–21%	0.105 Ω m	electrical	40 V	70	<30	*T*_m_ decrease with ς	(205)
PCL DMA	Fe_3_O_4_	2–12 wt %		magnetic	300 kHz, 5 W	43	20		(206)
PLA/PU (50/50)	CNT	1–5 wt %	0.33 Ω m for 5 wt %	electrical	50 V	50–70	<150	decrease *R*_R_ (13% smaller) in 5 wt %; faster shape recovery with electrical heating than conventional; higher *R*_R_ with electrical heating than conventional	(207)
EP	MWCNT nanopaper	40 wt %	3 × 10^–3^ Ω m	electrical	0–6 W	20–170	20	curing with resistive heating 105 °C after 40 s at 4.6 V; recovery at 76 °C	(97)
PE	CNF	0–11.6 vol%	0.1 Ω m for 11.6 vol%	electrical	1–40 V	100–110	100	*T*_c_ and elastic modulus increase with ς; *T*_m_ decrease with ς	(208)
PCL/MA (50/50)	MWCNT	0–10 phr	0.0384 Ω m	electrical	40 V	>70	56		(90)
PU	GNP			electrical	10–30 V	60 at 10 V	20		(209)
PCO/PE	MWCNT	8–15 vol%	0.0105 Ω m for 10 vol%	electrical	150 V	>120 after 2 min	90	triple SM effect; MWCNT dispersed in PCO; chemical cross-linking increases ρ_e_ due to agglomeration of fillers	(210)
SBS/LLDPE	CB	0–25 wt %	1.08 Ω m for 1 wt % CB; 7.4 × 10^–2^ Ω m for 25 wt % CB	electrical	0–120 V		60	elongation at break decrease by 50% from 0 to 25 wt % CB; tensile strength increase by >50%; recovery at 40 V	(211)
EP	rGO		0.0027 Ω m	electrical	1–6 V	35–240	5	bending	(110)
PEVA/PCL	MWCNT	5 wt %	0.0205 Ω m	electrical	20 V	97	12	recovery at 30 V	(212)
PU	CNT	10 to 50 layers	22.96 Ω m	electrical	40 V	90	30	CNT layers at different locations; *R*_R_ = 100% at 40 V; localized and selective actuation	(99)
EP/CE	GNP	0.8–2.4%	11.3 × 10^–2^ Ω m for 2.4%	electrical	20–120 V		98	strength increased by 25% for 1.6 wt %; recovery at 60 V	(213)
EP	Ag-CF	5.4 wt %, 1.8 wt %	95.1 Ω m for 5.4 wt %; 1 × 10^6^ Ω m for 1.8 wt %	electrical	60–120 V		60	*R*_F_ increase with ς; *R*_R_ decrease with ς	(81)
EP	MWCNT and CB	1.9 wt % total	2 × 10^5^ Ω m for 0.8 wt % MWCNT	electrical	225 V	>80	570	*R*_R_ = 99%	(214)
PLA/PU (70/30)	MWCNT and CB	3–5 phr CB and 0–2 phr MWCNT	<0.1 Ω m for CB 5 phr and >1 phr MWCNT	electrical	30 V	70 °C	100	70 °C after 100 s for 3 phr CB and 1 phr CNT; *R*_R_ = 0	(215)
PPDO–PCL/PU	Fe_3_O_4_	0–10 wt %		magnetic	7–30 kA/m at 256–732 kHz	65			(136)
PMMA–PEG	Modified Fe_3_O_4_	1–5 wt %		magnetic	100 kHz, 8 kW	40.4	59	*T* rise 4 °C for-surface modified Fe_3_O_4_ than for pristine; *R*_F_ = 90%; *R*_R_ = 95%	(216)
PCL	modified MWCNT	0–9 wt %	20 Ω m for >7 wt %	electrical	50 V for 5 wt %	70	120	decrease *R*_*R*_ with ς	(217)
PCL-Py	SWCNT	2 wt %	3.6 Ω m	electrical	50 V	65	20	increase *R*_R_ with ς	(104)
MA-based thermoset	Fe_3_O_4_	0–2.5 wt %		magnetic	0.33 mT at 297 kHz	50	40		(218)
PS	MWCNT and CB	1 wt % MWCNT and 10–15 wt % CB	0.03 Ω m for CB. 0.025 Ω m for CB+CNT randomly oriented. 0.0075 Ω m for CB+CNT chained	electrical	25 V	>95	75	MWCNT in chains, reduction of ρ_e_ by more than 1 order of magnitude; ρ_e_ increases with number of shape memory cycles	(107)
acrylate	NdFeB and Fe_3_O_4_	15 vol % of each		magnetic	40 mT at 60 kHz (to heat up Fe_3_O_4_) + 30 mT at 0.25 Hz to actuate beam	100 (and above) in 40 s		dual magnetic field activation; elastic modulus increases linearly with ς; *R*_F_ = 95%; *R*_R_ = 100%	(141)
PLA	Fe_3_O_4_	10–40 wt %		magnetic	30 kA/m at 268 kHz	>60	>200	nonmonotonic evolution of *R*_R_, modulus, tensile strength, and elongation at break with ς	(132)
Nafion	Fe_3_O_4_	15–25%		magnetic	Power 50–100%	internal *T* 50–110, surface *T* < 40	16	surface modification of Fe_3_O_4_ to improve distribution	(219)
PCL/PU	GNP	0–10 wt %	∼1 Ω m for 10 wt %	electrical	10–40 V	60 for 10 wt % at 10 V; 100 for 10 wt % at 30 V	60	5ecovery within 1 min for 7 wt % at 30 V; faster heating and recovery for higher ς; *T*_g_, χ_c_, viscosity increase with ς; *T*_m_and *T*_c_ decrease with ς	(220)
PLA	PPy		0.028 Ω m	electrical	15–40 V	120 for 40 V	2	microfiber membrane PPy coated; worse mechanical properties with Ppy ρ_e_ depends on polymerization time and temperature	(221)
PCLA	GO	4.86–13.29 vol %		electrical			2.5	*R*_R_ = 100%	(222)
PU/EP	G and CNT	0.8–3 wt % CNT and 2–12 wt % graphene	0.31 Ω m for 12 wt % graphene	electrical	100 V for 8% graphene and 3%CNT	60	150	compressive strain; densification at ε > 70%; 2% permanent deformation after 100 cycles	(223)

## Abbreviations

BMI: bismaleimide
BR: butadiene rubber
*C*_p_: heat capacity
CB: carbon black
CE: cyanate ester
CF: carbon fiber
CNF: carbon nanofiber
CNT: carbon nanotube
CSF: carbon short fiber
DMA: dimethacrylate
EOC: ethylene-1-octene copolymer
EP: epoxy
G: graphene
Gr: graphite
GO: graphene oxide
GNP: graphene nanoplatelets
LLDPE: linear low-density polyethylene
MA: methacrylate
MWCNT: multiwalled carbon nanotube
NGDE: neopentyl glycol diglycidyl ether
phr: parts per hundred rubber
PBS: poly(butylene succinate)
PCL: polycaprolactone
PCLA: poly(l-lactide-co-ε-caprolactone)
PCO: polycyclooctene
PDL: ω-pentadecalactone
PE: polyethylene
PEG: poly(ethylene glycol)
PEN: polyethylene naphthalate
PEVA: poly(ethylene-vinyl acetate)
PK: polyketone
PMMA: poly(methyl methacrylate)
PLA: poly(lactic acid)
PPC: poly(propylene carbonate)
PPDO: polydioxanone
PPy: polypyrrole
PS: polystyrene
PU: polyurethane
PVA: polyvinyl acetate
PVAl: poly(vinyl alcohol)
PVDF: polyvinylidene fluoride
Py: pyrene
*R*_F_: shape fixity ratio
rGO: reduced graphene oxide
*R*_R_: shape recovery ratio
SBS: poly(styrene-butadiene-styrene)
SMA: shape memory alloy
SMP: shape memory polymer
*T*: temperature
*T*_c_: crystallization temperature
*T*_g_: glass transition temperature
*T*_m_: melting temperature
*t*_R_: recovery time
ε: strain
κ: thermal conductivity
ρ_e_: electrical resistivity
ς: concentration
χ_c_: crystallinity
2W-SM: two-way shape memory
